# The optimal crowd learning machine

**DOI:** 10.1186/s13040-017-0135-7

**Published:** 2017-05-19

**Authors:** Bilguunzaya Battogtokh, Majid Mojirsheibani, James Malley

**Affiliations:** 1 Center for Information Technology, National Institutes of Health, Bethesda, MD USA; 2 Department of Mathematics, California State University Northridge, Northridge, CA USA

## Abstract

**Background:**

Any family of learning machines can be combined into a single learning machine using various methods with myriad degrees of usefulness.

**Results:**

For making predictions on an outcome, it is provably at least as good as the best machine in the family, given sufficient data. And if one machine in the family minimizes the probability of misclassification, in the limit of large data, then Optimal Crowd does also. That is, the Optimal Crowd is asymptotically Bayes optimal if any machine in the crowd is such.

**Conclusions:**

The only assumption needed for proving optimality is that the outcome variable is bounded. The scheme is illustrated using real-world data from the UCI machine learning site, and possible extensions are proposed.

## Background

The universe of statistical learning machines is still rapidly expanding, and new methods are being introduced almost daily. Despite these advances, choosing one machine over many other plausible machines, or, one particular version from within a family, can be arduous and resource intensive. Equally important, understanding how the schemes work and the results they produce, remains a separate and ongoing challenge. Unfortunately, for most researchers, many learning machines are “black boxes.” For a general, self-contained, and relatively nontechnical introduction to learning machines, see [1].

The scheme described here encourages the implementation of multiple and diverse machines. It begins with a family of machines, each making separate predictions or classifications, given the training data. These individual predictions are then used as inputs to a single machine. This final machine is itself functionally transparent, does not require any user-supplied tuning parameters or parameter estimation. It is virtually assumption free, as it only requires that the outcome variable is bounded.

Earlier version of this approach has been studied in the literature under the topic of *stacking*. More recently *deep learning* has been introduced, versions of which use individual machine predictions as layers, themselves used as inputs to another machine; for details of both, see [2].

The scheme discussed here is distinct from these approaches. Most notably, the optimal crowd uses predictions for a test point, generated by the separate machines, to direct the researcher to a specific subset of the training data. Then the *known* outcomes in the training data that are closest to the test point are simply averaged. As discussed below, when the scheme is used for pure classification, over zero/one outcomes, the measure of closeness is immediate, requiring no tuning or new parameters.

More precisely, the predictions of the separate machines are not averaged to generate a final summary value, so the scheme is not an ensemble or a committee method. Indeed, after the several machine predictions are used to sort the training data into small compartments in data space, the predictions are all effectively set aside. To repeat, the crucial property of this scheme is that, after the data partitioning, it averages over *known* outcomes in the training data rather than over the machine predictions for a test point.

It is not asserted that the learning machine discussed here will strictly outperform any single machine in the crowd, even in the limit of large data. However, given sufficient data, the method has been shown analytically to be at least as good as the best machine in the family; see [3]. For this reason the method is called here the *optimal crowd machine*. The technical basis of the particular method studied here, the optimal crowd machine, was presented in [4]; see also [5, 6, 7, 8, 3].

For any prediction or estimation problem, choosing a model, estimating parameters from data, or selecting kernel functions, for example, all introduce a small, or perhaps sizeable, cost to analysis and comprehension. Even before the predictions are assembled, a researcher must wade through these model selection decisions, all of which can be resource intensive and divert from understanding of the data itself, and the predicted outcomes… The optimal crowd machine makes none of these demands. In deploying a optimal crowd the researcher is free to reason over a diverse family of machines, using individual statistical preferences or subject-matter knowledge, and without having to make often difficult choices on model selection.

Equally important, in using the optimal crowd there is no declaration of a best model. There is no necessity for doing so, even assuming that they might be a single winning model in the family of machines. In the situation where one or more of the researcher-defined machines is, in fact, the best machine, the optimal crowd is provably as least as good as that model, given sufficient data.

This optimality, free of parametric assumptions, promotes the assembly of any collection of machines into a crowd, and, indeed, multiple families of machines are encouraged. For example, a suite of support vector machines can be proposed, using a wide range of researcher-chosen kernels. A collection of *k*-nearest neighbor’s schemes over a wide range of *k* values is also easily accommodated. And a collection of random forests with a wide range of terminal node sizes is another possible family of machines for the crowd.

The optimal crowd is studied here over a family of *k*-NNs, of random forests, and of SVMs. Machine versions within each family are all applied to the same data, and the optimal crowd uses the complete set of predictions for each test point as input data. Indeed, using the multiple machine predictions as new or, *syntheti*c features in the crowd allows the machines to learn from each other; see [9]. The particular machine families in this project are representative of machines often used in the data analysis community.

Most importantly, and distinct from these familiar machines, the crowd machine, by itself, requires no training data, and has no need for tuning parameters.

### Learning machines in a crowd

To demonstrate the scheme, several well-known families of learning machines were used, along with several versions within each family.

A *Random Forest* (RF) is a learning machine that consists of many classification, or regression, decision trees. A random bootstrap sample (sampling with replacement) of the data is used to grow a decision tree using selected features at each node of each tree. A node is split using a feature from the complete list of features that is locally best. Many splitting schemes are available, as are schemes for declaring the best split. The splitting is repeated until the tree reaches a terminal node sample size which is defined by the researcher. This process is repeated until a user-specified number of trees have been generated. Each test point is sent down all the trees, and the forest uses the majority vote across the collection of trees for the final prediction. In this study, just 100 trees were used, and the fraction of the input samples required to fill a terminal leaf node was varied;

A *k-nearest neighbor* (kNN) scheme uses training samples in a feature space and classification, or regression, is based on the distance of a specific test point to the training samples. For the *k* nearest points, the known outcomes in the training data are then averaged. In this study, several values for *k* were used;

A *support vector machine* (SVM) utilizes hyper planes in a transformed version of the sample data space to separate training data. This separation requires a specific, user-input kernel function, or weighting function, such that a so-called margin of a support vector scheme is maximized. In this study, several kernel functions were used;

*Synthetic learning machines* were also used in this project; see [9]. Here, each prediction of each machine is generates a new, *synthetic* feature, and each feature is then added to the original, training data. In the data analysis below, the synthetic features were also input to a single random forest learning machine, and the output from this machine was also added to the original data. In this way, learning machines can learn from each other; see the Discussion below.

### The optimal crowd classifier

The optimal crowd classifier provides a solution to the problem of pure classification, where the independent outcome is just zero/ one, or, case/control outcomes such as {tumor, not tumor}. The individual machines in the crowd are each taught from the original training data and return zero/one predictions for each point.

As mentioned above, the data can also be supplemented to include synthetic features. For this, the prediction of each machine at each point in the training data is added as a new feature to the original data. In this study, results from the optimal crowd were studied with and without the inclusion of synthetic features.

Individual machine predictions are merely used to guide the researcher to known outcomes that are given in the training data, and these are then averaged for a final prediction of a test point. Notably, this method does not average over multiple machine predictions but rather averages over *known* outcomes in the given data.

**Fig. 1:**
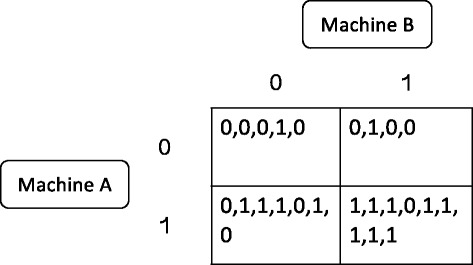
In this 2 × 2 table, the labels for the rows and columns refer to the predictions made by Machine A and Machine B on all the training data. The zeroes and ones in the four cells are *known* outcomes in the data, and the optimal crowd directs the analysis to these outcomes

An example can help make this more transparent, and refer to Fig. 1. In this 2 × 2 table, the zero and one labels for the rows and columns refer to the predictions made by Machine A and Machine B on all the training data. The zeroes and ones are known outcomes and the optimal crowd directs the analysis to certain of these outcomes.

For example, suppose that for a single test point, Machine A predicts 0, and Machine B predicts 1. These predictions direct the researcher to the upper right corner in this table. The optimal crowd machine averages over the known values of training data in this cell. By inspection, the optimal crowd machine estimates the probability for that test subject as 3/4 for being Outcome 0, and 1/4 for being Outcome 1. For the final classification, using a 50% cut-point, the test point is declared as Outcome 0.

If there were no outcomes in this cell, the prediction for the test point would move on to the other available machine predictions. Thus, a {0, 1} test point prediction for the two machines is one unit away from both the {0, 0} and {1,1} cells, and two units away from the {1,0} cell. Therefore the optimal crowd would average over the known values in the {0,0} *and* the {1,1} cells, assuming machine predictions from the training data led to either of these cells.

Hence, in the optimal crowd scheme, there is never a possible empty cell problem. More precisely, the machines might not make all possible pairs of feasible predictions over the training data, but they don’t need to--only the filled cells made by the pair of machines on the training data are used in the final result.

An optimal crowd can incorporate either pure classification or probability machines, or both. The output from a *probability machine* is converted to a simple classification using a 50% cut-point. Both types of machines are used in this project; see [10] for more on probability machines.

### Datasets

Two datasets were studied in this project.

First, the Wisconsin Breast Cancer Data Set (Original) was obtained from the Machine Learning Repository of the University of California, Irvine [http://archive.ics.uci.edu/ml/]. The data set consisted of 10 features that have real integer values from 1–10 with 699 instances. These features describe characteristics of cells that may or may not be cancerous. The original dataset contains 16 instances of a missing feature values. Samples with missing values were deleted from the data set, reducing the sample size to 683: 444 instances were benign, classified as 0, and 239 instances were malignant, classified as one. One hundred fifty samples from each class were randomly chosen to comprise the training data while fifty samples from each class were taken to comprise the testing data.

And second, the SpamBase Data set, was also obtained from the Machine Learning Repository of the University of California, Irvine [http://archive.ics.uci.edu/ml/]. The data set consisted of 57 features that are either continuous real or continuous integer values with 4601 instances; 2788 instances were not spam, classified as 0, and 1813 instances were spam, classified as one. A total of 1,350 samples from each class were randomly chosen to form the training data, while a separate 450 samples were randomly chosen to form the testing data. The features (predictors, independent variables) described characteristics of text taken from actual emails that may or may not be spam.

Finally, features in both datasets were scaled to be in the range [0,1]. For the purpose of using the optimal crowd on a variety of machine learning families were deployed.

## Results

Percent error was used to evaluate machine performance, that is, the total number of samples misclassified by a machine divided by the total number of samples in the data and a five-fold cross-validation (5XCV) was undertaken.

**Fig. 2:**
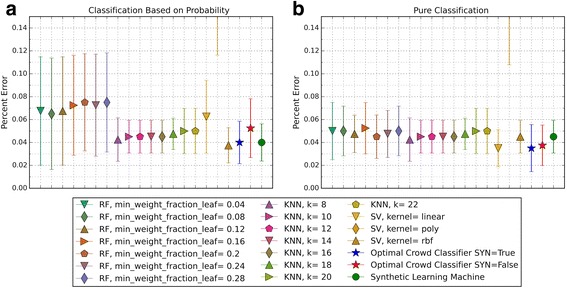
Depicts 5XCV outcomes on 18 learning machines used in the optimal crowd for Breast Cancer Data. 200 case and 200 control samples were used for the 5XCV. The percent errors of each fold were averaged for a final result. The error bars represent one standard error. The label “SYN = True” means that synthetic features were used in the optimal crowd, and the label “SYN = False” refers to the lack of synthetic features. Panel **a** depicts machine performances for classification based on probability, and Panel **b** depicts machine performances for pure classification

**Fig. 3:**
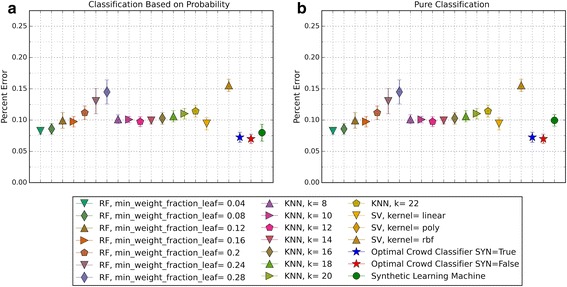
Depicts 5XCV outcomes on 18 learning machines used in the optimal crowd classifier for SpamBase Data. 1800 spam and 1800 not-spam samples were used for the 5XCV. The percent errors of each fold were averaged for a final result. The error bars represent one standard error. The label “SYN = True” means that synthetic features were used in the optimal crowd, and the label “SYN = False” refers to the lack of synthetic features. Panel **a** depicts machine performances for classification based on probability, and Panel **b** depicts machine performances for pure classification

**Fig. 4:**
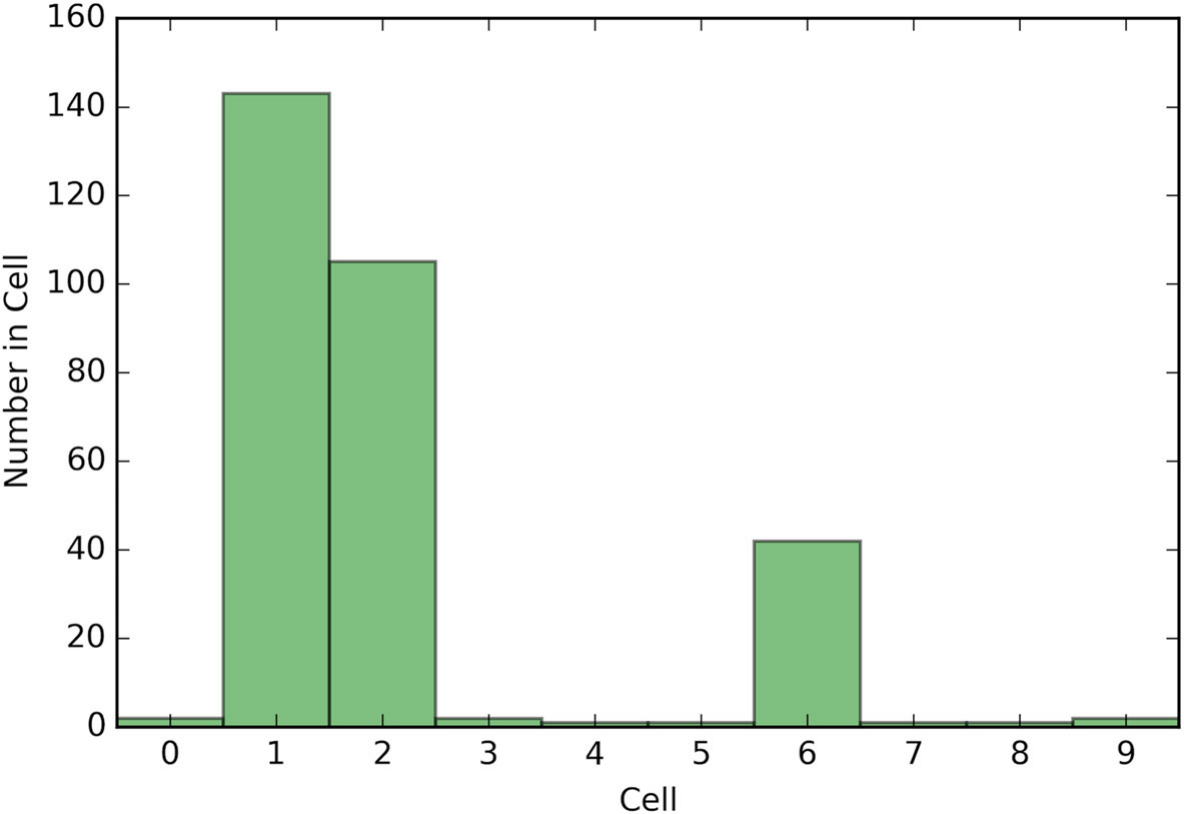
Depicts a histogram for cell counts of known outcomes on Breast Cancer data using the optimal crowd with 18 machines and no synthetic features

**Fig. 5:**
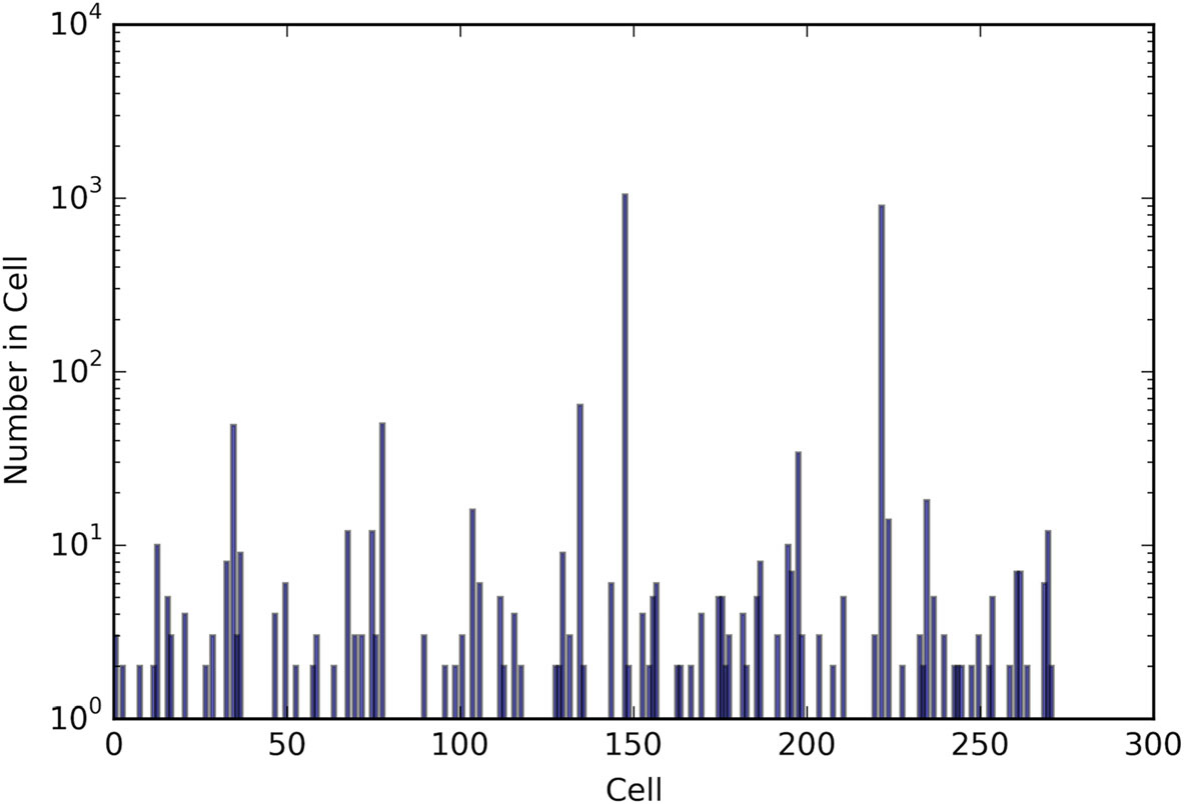
Depicts a histogram for cell counts of known outcomes on SpamBase data using the optimal crowd with 18 machines and no synthetic features

**Fig. 6:**
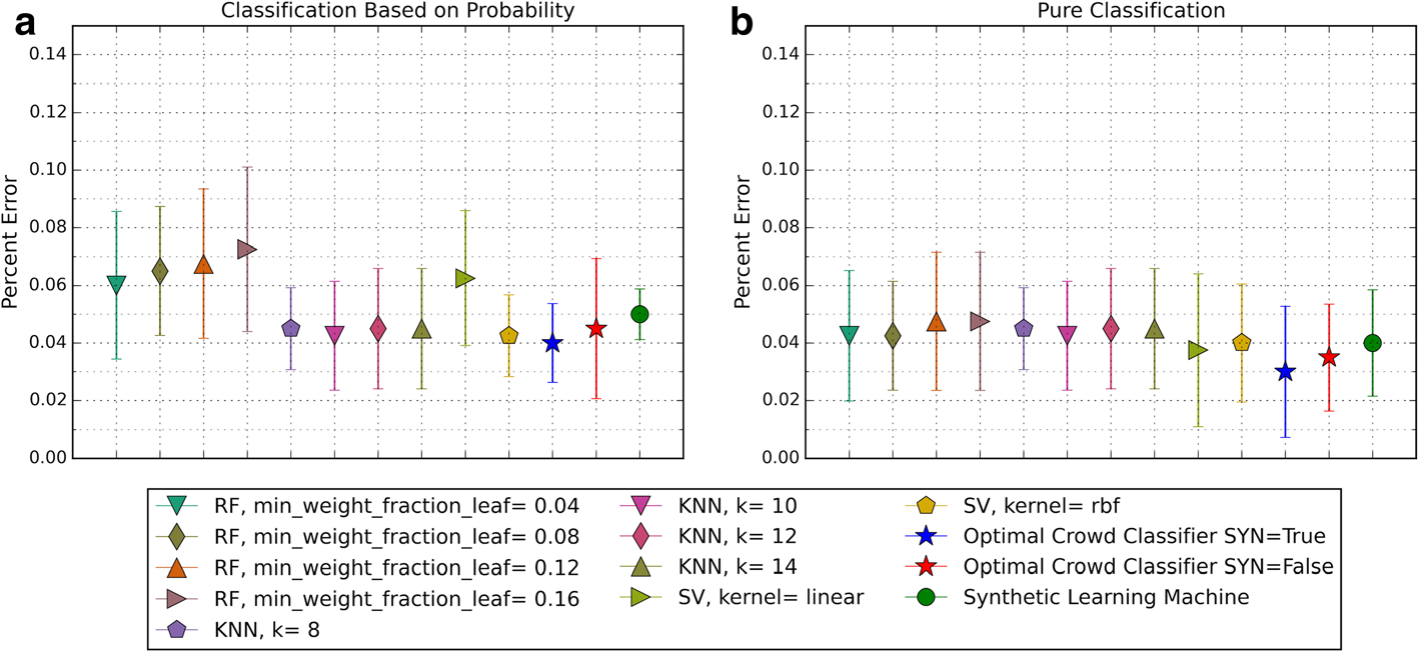
Depicts 5XCV outcomes on ten learning machines used in the optimal crowd for Breast Cancer Data. 200 case and 200 control samples were used for the 5XCV. The percent errors of each fold were averaged for a final result. The error bars represent one standard error. The label “SYN = True” means that synthetic features were used in the optimal crowd, and the label “SYN = False” refers to the lack of synthetic features. Panel **a** depicts machine performances for classification based on probability, and Panel **b** depicts machine performances for pure classification

**Fig. 7:**
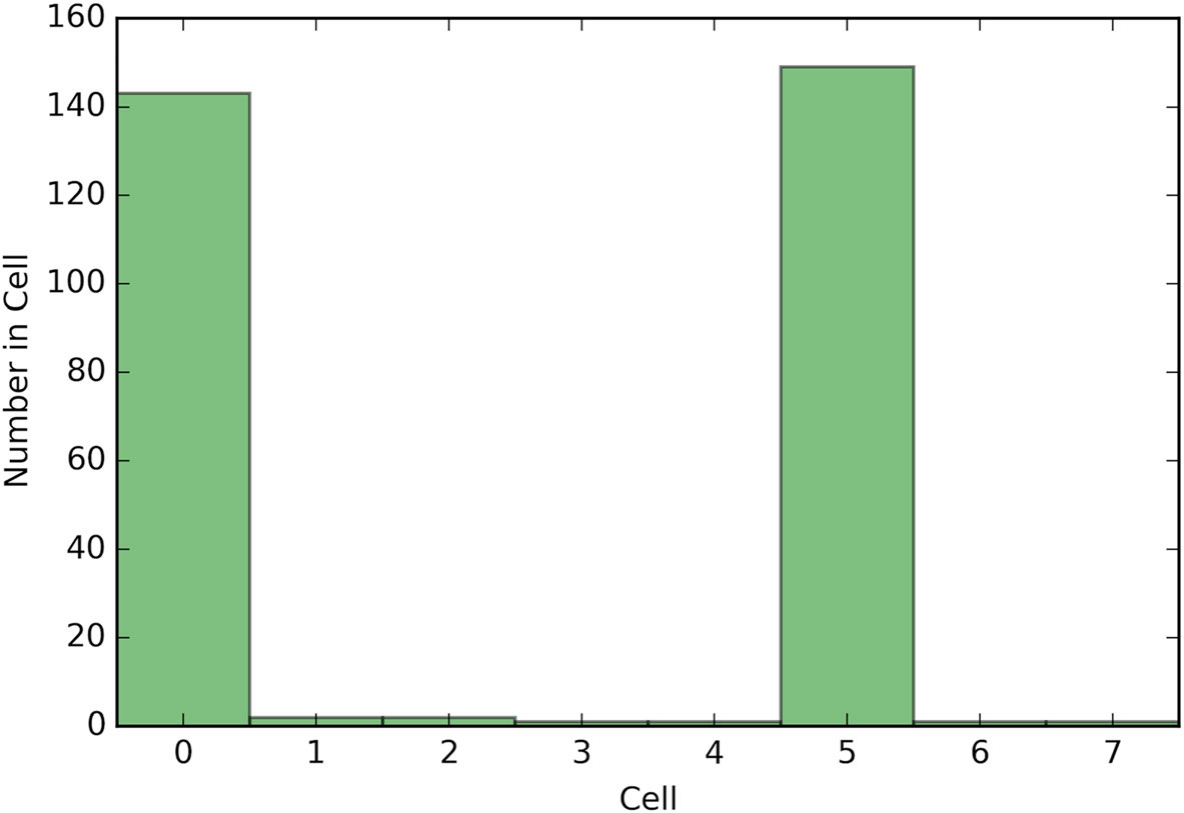
Depicts a histogram for cell counts of known outcomes on Breast Cancer data using the optimal crowd with 10 machines and no synthetic features

**Fig. 8:**
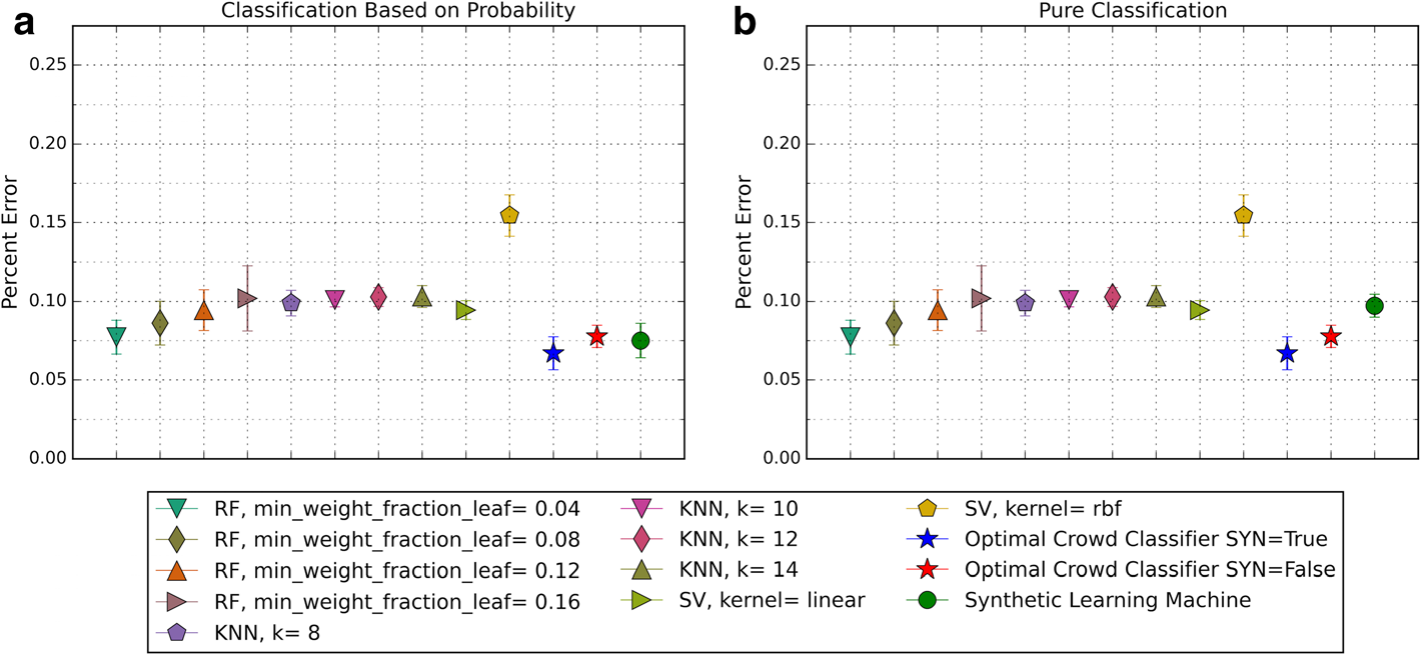
Depicts 5XCV outcomes on 10 learning machines used in the optimal crowd classifier for SpamBase Data. 1800 spam and 1800 not-spam samples were used for the 5XCV. The percent errors of each fold were averaged for a final result. The error bars represent one standard error. The label “SYN = True” means that synthetic features were used in the optimal crowd, and the label “SYN = False” refers to the lack of synthetic features. Panel **a** depicts machine performances for classification based on probability, and Panel **b** depicts machine performances for pure classification

**Fig. 9:**
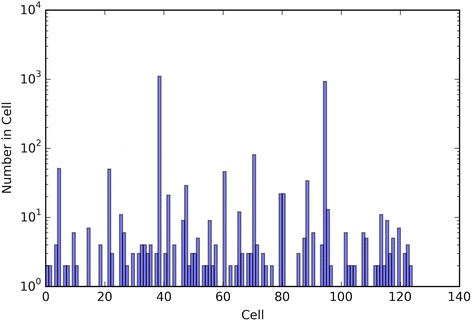
Depicts a histogram for cell counts of known outcomes on SpamBase data using the optimal crowd with ten machines and no synthetic features

**Fig. 10:**
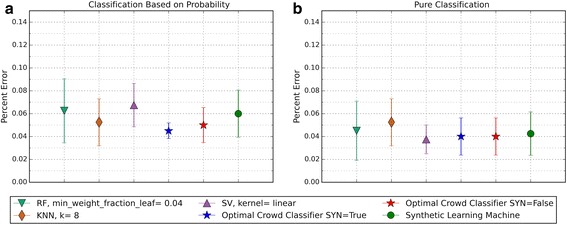
Depicts 5XCV outcomes on three learning machines used in the optimal crowd for Breast Cancer Data. 200 case and 200 control samples were used for the 5XCV. The percent errors of each fold were averaged for a final result. The error bars represent one standard error. The label “SYN = True” means that synthetic features were used in the optimal crowd, and the label “SYN = False” refers to the lack of synthetic features. Panel **a** depicts machine performances for classification based on probability, and Panel **b** depicts machine performances for pure classification

**Fig. 11:**
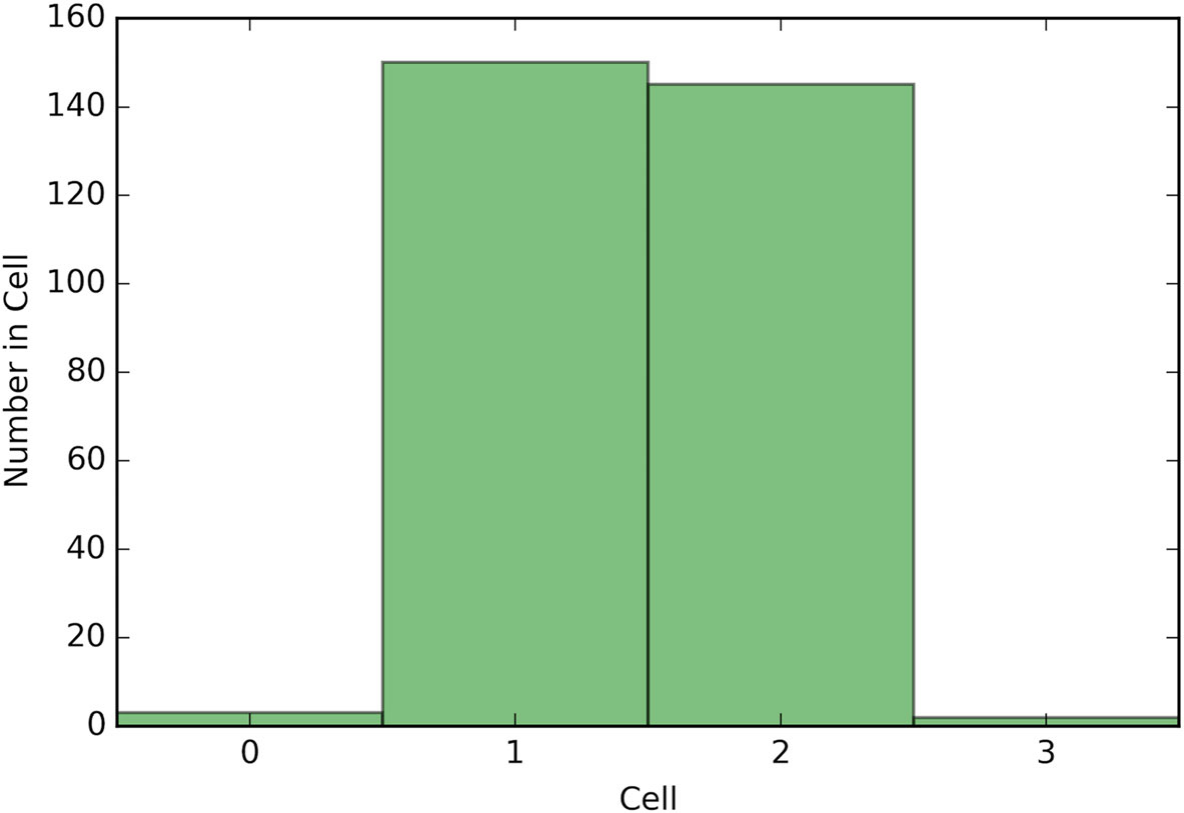
Depicts a histogram for cell counts of known outcomes on Breast Cancer data using the optimal crowd with 3 machines and no synthetic features

**Fig. 12:**
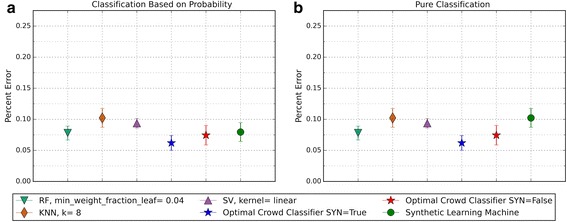
Depicts 5XCV outcomes on 3 learning machines used in the optimal crowd classifier for SpamBase Data. 1800 spam and 1800 not-spam samples were used for the 5XCV. The percent errors of each fold were averaged for a final result. The error bars represent one standard error. The label “SYN = True” means that synthetic features were used in the optimal crowd, and the label “SYN = False” refers to the lack of synthetic features. Panel **a** depicts machine performances for classification based on probability, and Panel **b** depicts machine performances for pure classification

**Fig. 13:**
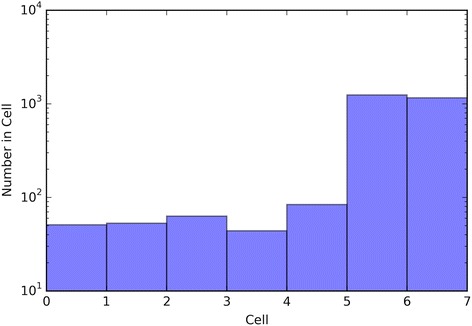
Depicts a histogram for cell counts of known outcomes on SpamBase data using the optimal crowd with 3 machines and no synthetic features

The performance of the optimal crowd and the individual machines were applied to pure classification and classification based on probability, where the output from a probability machine is converted to a simple classification using a 50% cut-point. Figures 2, 3, 6, 8, 10, and 12 give the 5XCV results of applying both versions, with and without synthetic features, of the optimal crowd and individual machines on the Breast Cancer and SpamBase datasets for varying numbers of individual machines included in the optimal crowds. The numbers of learning machines tested were eighteen, ten, and three. Note that {SVM kernel = poly} is not shown on some graphs since the percent error exceeded 0.50 in some instances. Additionally, for each performance result, a histogram depicted the number of training instances (known outcomes) for each cell in the optimal crowd is included to examine if large disparities in distribution across the cells and a large number of cells would negatively impact performance in the optimal crowd; these histograms are Figs. 4, 5, 7, 9, 11, and 13.

For both pure classification, and for classification using a probability machine, and on both data sets, the optimal crowd classifiers do quite well. The optimal crowd requires no training sample as do many other machines. Hence, for example, given only the 400 subjects in Breast Cancer Data (200 per group), the 5XCV process applied to the optimal crowd, is at each step, given just 20% of the data, that is about 40 subjects from each group (80 in total). To repeat, the optimal crowd is making its predictions using the results of the many machines as they are applied to just a fraction of the complete data.

It is noted that the optimal crowd performs well regardless of the number of machines in the crowd. The crowd is analytically indifferent to the number of machines. From the cell count graphs, it is clear that some cells are sparsely populated when greater numbers of machines are utilized. However, this does not necessarily indicate that the cell provides a weak estimate, and our results as above demonstrate this; see Figs. 4, 5, 7, 9, 11, and 13. Indeed, the analytical results of [3] do not depend on the number of machine for optimality, though, like any statistical procedure, closeness to the Bayes minimum error is a function of sample size.

## Discussion

Not shown above, in Figs. 2 and 3, is that some probabilities generated by probability machines for certain test instances are not in the unit interval. Specifically, these were out of bounds probabilities generated by SVM predictions for these two test cases. Probabilistic, or regression estimates that were not in the interval [0, 1] are a known problem of SVMs. For our study, we used a simple rule: estimated SVM “probabilities” larger than 1 were mapped to 1, and those less than 0 were mapped to 0.

The optimal crowd has been rigorously shown to be at least as good as the best machine in the family, as the sample size increases; see [3]. Indeed, [3] demonstrated optimality for the crowd operating as a regression machine. And the single assumption guaranteeing this optimality in either case is that the known outcomes are bounded; see the discussion following Proposition 2.2 in [3]. No other conditions are placed on the data, and indeed nothing is assumed for the original measured variables, from which the separate machines derive their predictions.

As with any statement of statistical optimality relating to sample size, the amount of data required to well-approximate the Bayes error rate lower bound, will vary from problem to problem. The posted error rates of the optimal crowd for the 5XCV analysis on each data set support the premise that the crowd performs well on small and medium sample sizes.

Furthermore, it has been formally demonstrated that the rate of convergence is not a function of the dimensionality of the data; see the discussion in [3] following *Theorem* 2.1. Hence, the curse of dimensionality, at least relative to convergence rates, is not a problem of the optimal crowd machine.

The optimal crowd can be applied to estimation of risk effects, or log odds, or risk differences, for each test subject. Typically, some version of a logistic regression model is proposed for the data, but this also requires parameter estimation and tuning, as with any classical statistics model-fitting schemes. A regression version of the optimal crowd can easily accommodate a collection of methods or models or machines that separately estimate risk effects. The use of multiple learning machines acting as probability estimation scheme, for estimation of risk effects for each subject, was examined in [11]. The application of the optimal crowd to such estimation will be considered elsewhere.

Along the same lines, multiple Bayes models with different priors can be sent to the optimal crowd. If one of the models and its associated priors is correct, then given sufficient data, the optimal crowd will converge to the predictions from this correct model. This offers a transparent resolution of the Bayes consensus problem.

Moreover, the many layers of a neural net as commonly used in Deep Learning can be put together in any combination as individual machines in a crowd. Given sufficient data, the crowd will optimize over the entire collection of Deep Learning models.

Finally, it is notable that by using synthetic features, as mentioned above, the optimal crowd allows individual machines in the family to learn from each other. And this is another topic for future work. For further information, refer to additional notes in [12].

## Appendix 1

The Python libraries SciPy and scikit-learn were used in this project.

## References

[1] Malley JD, Malley KG, Pajevic S. Statistical learning for biomedical data. Cambridge University Press, Cambridge, UK; 2011.

[2] For details of stacking see, for example: http://stats.stackexchange.com/questions/18891/bagging-boosting-and-stacking-in-machine-learning; and for details of deep learning see, for example: http://deeplearning.net/. Accessed 1 Apr 2017.

[3] Biau G, Fischer A, Guedj B, Malley JD (2016). COBRA: a combined regression strategy. J Multivar Anal.

[4] Mojirsheibani M (1997). A consistent combined classification rule. Stat Probab Lett.

[5] Mojirsheibani M (1999). Combining classifiers via discretization. J Am Stat Assoc.

[6] Mojirsheibani M (2002). An almost surely optimal combined classification rule. J Multivar Anal.

[7] Balakrishnan N, Mojirsheibani M (2015). A simple method for combining estimates to improve the overall error rates in classification. Comput Stat.

[8] Mojirsheibani M, Kong J (2016). An asymptotically optimal kernel combined classifier. Stat Probab Lett.

[9] Ishwaran H, Malley JD (2014). Synthetic learning machines. BioData Mining.

[10] Malley JD, Kruppa J, Dasgupta A, Malley KG, Ziegler A (2012). Probability machines: consistent probability estimation using nonparametric learning machines. Methods Inf Med.

[11] Dasgupta A, Szymczak S, Moore JH, Bailey-Wilson JE, Malley JD (2014). Risk estimation using probability machines. BioData Min.

[12] For further notes on the optimal crowd learning machine: http://bit.ly/2k1g6bk. Accessed 1 Apr 2017.

